# Challenging drug-resistant TB treatment journey for children, adolescents and their care-givers: A qualitative study

**DOI:** 10.1371/journal.pone.0248408

**Published:** 2021-03-10

**Authors:** Mrinalini Das, Taanya Mathur, Shilpa Ravi, Augusto C. Meneguim, Aparna Iyer, Homa Mansoor, Stobdan Kalon, Farah Naz Hossain, Shrikala Acharya, Gabriella Ferlazzo, Petros Isaakidis, Harshad P. Thakur

**Affiliations:** 1 Médecins Sans Frontières, Mumbai, India; 2 Tata Institute of Social Sciences, Mumbai, India; 3 Mumbai Districts AIDS Control Society, Mumbai, India; 4 Southern Africa Medical Unit, Médecins Sans Frontières, Cape Town, South Africa; 5 National Institute of Health and Family Welfare, New Delhi, India; Maastricht University, NETHERLANDS

## Abstract

**Background:**

Childhood multidrug-resistant TB (MDR-TB) still affects around 25000 children every year across the globe. Though the treatment success rates for drug-resistant TB (DR-TB) in children are better than adults, children and adolescents face unique hurdles during DR-TB (MDR-TB, Pre-XDR TB and XDR-TB) treatment. This study aimed to understand the patients, guardians and healthcare providers’ perspectives about DR-TB treatment journey of patients and caregivers.

**Methods:**

This is a qualitative study involving in depth-interviews of purposively selected adolescents (n = 6), patients guardians (for children and adolescents, n = 5) and health care providers (n = 8) of Médecins Sans Frontières (MSF) clinic, Mumbai, India. In-depth face to face interviews were conducted in English or Hindi language using interview guides during September-November 2019. The interviews were audio-recorded after consent. Thematic network analysis was used to summarize textual data. ATLAS.ti (version 7) was used for analysis.

**Result:**

The age of adolescent patients ranged from 15–19 years and four were female. Five guardians (of three child and two adolescent patients) and eight healthcare providers (including clinicians- 2, DOT providers-2, counselors-2 and programme managers-2) were interviewed. The overarching theme of the analysis was: Challenging DR-TB treatment journey which consisted of four sub-themes: 1) physical-trauma, 2) emotional-trauma, 3) unavailable social-support and 4) non-adapted healthcare services. Difficulties in compounding of drugs were noted for children while adolescents shared experiences around disruption in social life due to disease and treatment. Most of the patients and caregivers experienced treatment fatigue and burnout during the DR-TB treatment. Participants during interviews gave recommendations to improve care.

**Discussion:**

The TB programmes must consider the patient and family as one unit when designing the package of care for paediatric DR-TB. Child and adolescent friendly services (paediatric-formulations, age-specific counselling tools and regular interaction with patients and caregivers) will help minimizing burnout in patients and caregivers.

## Background

Childhood drug-resistant TB (DR-TB) is a neglected illness across the globe. Though the precise burden of childhood multidrug-resistant TB (MDR-TB) is still unknown, the global estimates for MDR-TB in children is around 25,000–32,000, of whom 21% probably die due to disease [1].

Multiple discussions have gained momentum for children with DR-TB around improved diagnostics, shorter treatment duration and injectable-free, palatable paediatric formulations [2,3]. The national TB programmes have been taking steps for improved care for childhood DR-TB [4], however measures for optimum care for children and adolescents with DR-TB [with focus on MDR-TB, pre-extensively drug-resistant TB (Pre-XDR TB) and extensively drug-resistant TB (XDR-TB)] still needs attention [5,6]. Adolescent age group (10–19 years) have their distinct challenges, however, their needs get incorporated along with children (10–14 years) and adults (15 years and above) [7,8]. Focus on this specific age group is required so that age appropriate care packages and policy recommendations can be developed for them.

Although the treatment outcomes for DR-TB are reported to be better in children than adults [9,10], the paediatric and adolescent population has unique developmental needs (search for identity, autonomy [7], social support, peer-relationships) and DR-TB disease and treatment might have a more profound impact in their lives than it would in the lives of adults. While children need more family care and support, adolescents often demand autonomy. Children and adolescents with DR-TB often face social isolation, stigma, depression, and poor self-esteem [7]. These impacts could be similar to other serious illnesses like HIV [11], while some are not only related to DR-TB disease but also on the painful and challenging DR-TB treatment [12–14]. Adolescents, more than children, often report about long-lasting internalized stigma after DR-TB treatment, thus needs attention [15].

In India, Médecins Sans Frontières (MSF) has been providing treatment and care to patients with drug-resistant TB (DR-TB) including children and adolescents in Mumbai- one of the known DR-TB hotspots in the country [16–18]. To bridge the evidence gap in knowledge of children and adolescents’ experiences during DR-TB (including MDR-TB, PreXDR-TB, XDR-TB) treatment, this qualitative study was conducted to understand the perspectives of adolescents receiving DR-TB treatment, guardians (of children and adolescents with DR-TB) and healthcare providers about DR-TB treatment journey of children, adolescent and their caregivers.

## Methods

### Study design

This was a qualitative study involving in depth-interviews of adolescent patients, guardians (of children and adolescents with DR-TB) and health care providers.

### Setting

MSF clinic has been providing ambulatory, free of charge DR-TB treatment in Mumbai since 2006 [19]. The clinic provides sample collection facilities for children (gastric aspiration with nasogastric tube, sputum induction) or refers the children to nearest hospital for sample collection (gastric lavage, fine-needle aspiration cytology) which is sent for testing to an accredited laboratory (GeneXpert, Line probe assay, Culture-Drug susceptibility test (C-DST)) for DR-TB. Once diagnosed, patients receive DR-TB treatment based on C-DST results and drugs exposure.

A multi-disciplinary team including doctors, nurses, counsellor, peer educators provide treatment and care to the patients. The team provides information about DR-TB disease and treatment (including adverse events due to drugs and monthly culture follow up) to patient and their family at the time of initiation and repeated during treatment. The patients receive nutritional support during the DR-TB treatment. One family member (mother or father) is identified as a ‘care buddy’ for children and adolescents, who closely provides psycho-social support and helps in treatment adherence. Directly observed treatment (DOT) at their home is carried out for first six months by outreach nurses. After six months, the treatment is supervised by the identified care buddy in the family. Caregivers of young children (0–5 years) receive training for dose modification (or drug compounding) by clinic pharmacists before treatment initiation and later supervised by outreach nurses at their homes. Tailor made counselling and information about the disease and treatment is provided to patients (age-appropriate individualised counselling adapted to children and adolescents, flipchart and cartoon based information-education-communication materials, separate sessions with guardians of children and adolescents, close follow up with the patients). A self-administered monthly checklist mentioning daily medications is used for monitoring treatment adherence for the patients. The checklist is completed by adolescents and guardian of children aged less than 10 years old and reviewed with counsellor during routine follow up every month.

### Study population and participants

Purposive sampling was used to recruit the study participants. Adolescents (15–19 years, n = 6) who had received more than one year of DR-TB treatment or were cured (within 6 months) at the time of interview, were included in the study. Along with adolescents, guardians (n = 5) of children (0–5 years and 6–9 years) and adolescents (10–19 years) receiving DR-TB treatment, health care providers caring for DR-TB patients (n = 8) were included. The guardians were interviewed as a proxy for detailing experiences of children (0–5 and 6–9 years), and how their own life was also influenced by DR-TB treatment of their children. Healthcare providers (who provided direct treatment and care to patients for more than two years: clinicians, DOT providers and counsellors) and programme managers (responsible for overall implementation and monitoring of TB programme for more two years) were included in the study population to describe their experiences when delivering care to children and adolescents with DR-TB and how they perceived challenges faced by this vulnerable group.

### Data collection

In-depth face to face interviews were conducted by the principal investigator (MD) using an open-ended interview guide, in English and preferred local language (Hindi) between September and November 2019. Separate interview guides were used to interview patients, family members, health care providers and programme managers (S1 Appendix). The main topics covered in the interview were related to experiences during diagnosis, treatment, routine daily activities during treatment, episode of adverse events, how they maintained adherence, episodes of missing doses, treatment adherence checklist, healthcare providers involvement in treatment and care. The identified participants (adolescents and guardians) were first given information about the study (objective of the study, what would be expected from the participants) over the telephone by the clinic counsellors. The participants who were willing to discuss further about the study, were requested for an appointment to discuss about the study and consent process in detail. The interviews with participants (who gave consent for the study) were conducted in a closed separate room, in the treatment facility. Infection control measures were considered for the interviews. The principal investigator was a female researcher, had a Master’s in public health (MPH), fluent in English and local languages, and trained in qualitative research. Though the interviewer was an employee of the organization, she was not directly involved in patient care. All the interviews were carried out as per availability of study participants. The interviews were audio recorded after obtaining informed consent. The median (min-max) for time of interviews was 30 (13–60) min. At the end of each interview, the interviewer shared the summary of findings for participant validation.

### Data management and analysis

The interview transcripts were prepared within one week of the interview. We utilized thematic network analysis as our framework for analysis, as described by Attride-Stirling [20]. Thematic analysis summarizes textual data by organizing the main themes and their linkages in a web-like network (known as thematic network). The thematic network facilitates the structuring and depiction of main themes in one nexus [20]. Data was entered in ATLAS.ti (version 7) for analysis. Two co-authors (MD and TM) coded each interview transcript independently. Once the individual list of codes was prepared, the two authors together identified the similar codes. The dissimilar codes were discussed within the co-authors (based on the reviewing the narratives together) and a consensus was reached to prepare the final list of codes. Thereafter, the codes with common sub-themes and themes were grouped together and the network web was prepared. The findings were reported by using ‘Consolidated Criteria for Reporting Qualitative Research’ (COREQ guideline) [21].

### Ethics

The study received ethics approval from Ethics Review Board of Médecins Sans Frontières, Geneva, Switzerland (MSF ID #1928, dated 12 September 2019) and Institutional Review Board of Tata Institute of Social Sciences, Mumbai, India (Serial No. 2018-19/19, dated 19 June 2019). For participants more than 17 years of age, informed written consent was taken from study participants. For adolescents (aged 10–17 years), in addition to their assent for participation, consent was taken from their guardians for their participation.

## Results

### Participant characteristics

Of the six adolescents who participated in the interview, four were females. The age of the patients ranged from 15–19 years. Four had completed more than 12 months of treatment, while one was cured, and the other had completed treatment at the time of interview. A total of five guardians [of three child (aged 3, 8, 9 years) and two adolescent patients (aged 12 and 15 years) each] were interviewed, among whom four were mothers while one was the father of the patient. Of the guardian group, three children and two adolescents, three (2-child and 1-adolescent) patients were cured at time of interview, while two had completed more than 12 months of treatment. All the patients and caregivers lived in slums of known high DR-TB burden areas.

All children and adolescent patients included in the study (either as participants or their guardians, n = 10; one was common) received DR-TB treatment including Bedaquiline (BDQ) and/or Delamanid (DLM). Six had history of previous TB treatment, and all of them received injectable during previous TB treatment. In the current treatment regimen, six received injectable (three received either Amikacin, Kanamycin or Capreomycin, two received Imipenem/Meropenem, while one received concomitant Imipenem and Amikacin). Of these six, three patients received injectable both during current and previous treatment. Only one patient (aged 3 years) did not have exposure to injectable. All ten patients received Clofazamine while seven received Cycloserine during the treatment. The median (min-max) duration of treatment in patients who completed treatment at the time of interview (n = 5) was 21(20–22) months.

Eight healthcare providers (including clinicians- 2 DOT providers-2, counselors-2 and programme managers-2) were interviewed. All the healthcare providers had an experience of more than two years in providing care to children and adolescents with DR-TB.

### Challenging DR-TB treatment journey

**Fig 1:**
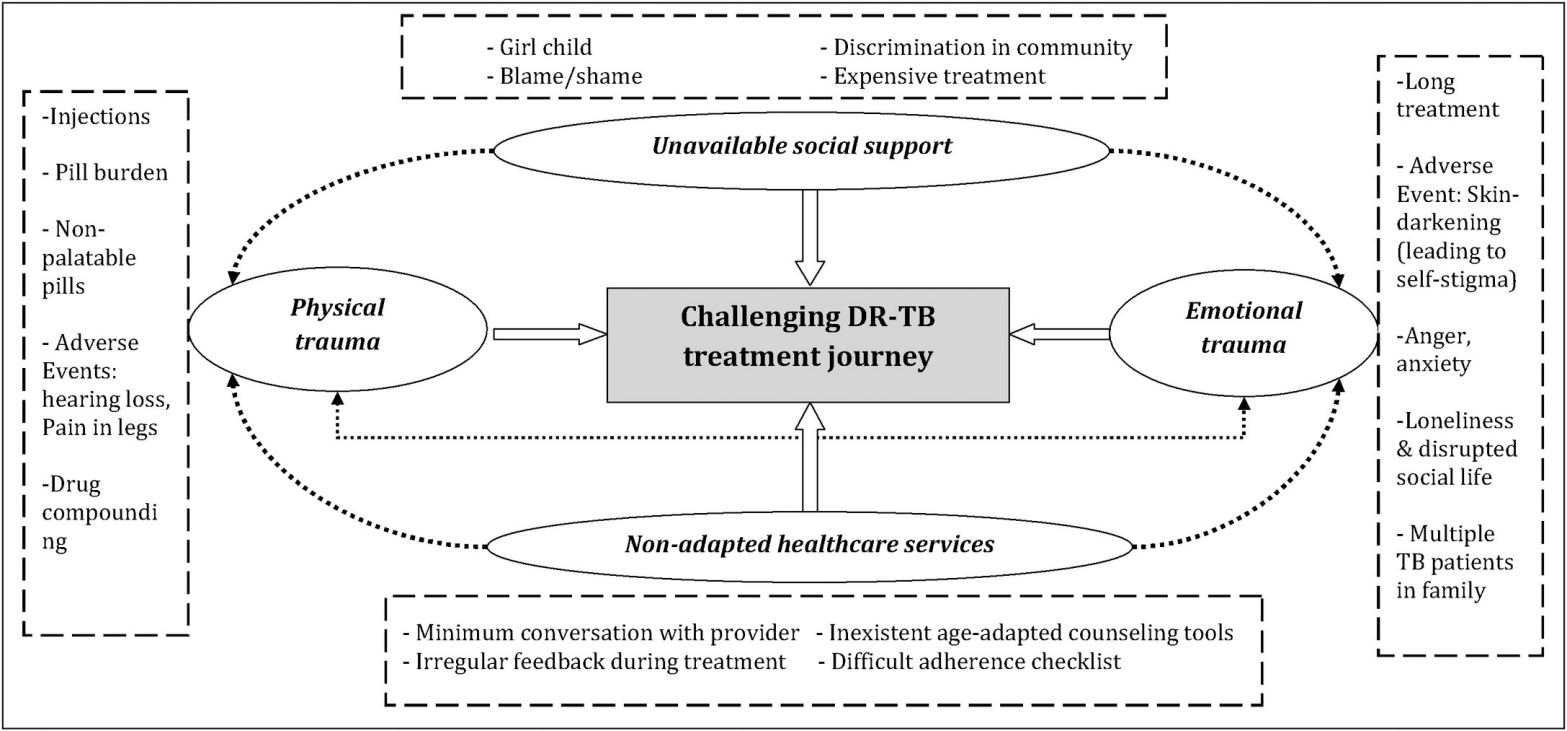
Non-hierarchical thematic network for ‘challenging DR-TB treatment journey’ in children and adolescents, Mumbai, India, 2019.

The overarching theme of our qualitative analysis was: Challenging DR-TB treatment journey. The overarching theme consisted of four sub-themes: 1) physical trauma, 2) emotional trauma, 3) unavailable social support and 4) non-adapted healthcare services. The thematic analysis in a non-hierarchical network is shown in Fig 1.

#### 1) Physical trauma

*1*.*1*. *Injections*. Injectable during treatment was the most painful experience for patients and guardians, common for those who received second-line injectable (Amikacin, Kanamycin or Capreomycin). Patients and care-givers expressed moments of soreness and bleeding at the injection site when the patients received injectable every day. The experiences with injectable haunted the patients even after their treatment finished.

*“The time I received injections was very difficult for me. I could not sit properly those days. There used to be a big swelling at the injection site, my mother had to rub ice on it all night. I could not sleep because of the pain.” (Patient 4; 16 year female)*.

*“Injection was the worst during treatment. I can have multiple medicines. But it used to bleed daily due to injection. I don’t want injections anymore.” (Patient 1; 15 year female)*.

“When injections were going on, it was very painful; he used to cry a lot. He used to say that now that my cough is better, why do I need injections. It used to bleed sometimes. Twenty eight injections were given to him… that was the most painful moment for me.” (Guardian of 12 year male).

*1*.*2*. *Pill burden*. Patients and caregivers complained about the multiple drugs patients had to take in a day. Healthcare providers described that the drugs given for adverse events during DR-TB treatment increased the pill burden of DR-TB medications, thus patients had to take around 15–20 pills per day. Guardians and DOT providers felt it took a lot of time for patients to take their pills, especially children of younger age group (0–5 years). Pill burden also increased as paediatric formulations were not available for many drugs. The mere sight of multiple drugs made them feel nauseous.

“I had to take 15–20 medicines in one day, 4–5 were for side effects only” (Patient 1; 15 year female).

*1*.*3*. *Non-palatable pills*. Taste and smell of few drugs were unbearable for many patients. Though the nausea and vomiting improved during the course of treatment, but Ethionamide was most difficult to take for the patients.

*“The most bitter medicine was given at the end, because often she used to vomit out… that medicine was like a poison.” (Guardian of 3 year female)*.

“Ethionamide has a strong smell, tablet causes gastritis… kids see the tablet and start vomiting. When you cut the tablet, it gives more nausea and vomiting.” (Healthcare provider 2).

*1*.*4*. *Adverse events during DR-TB treatment*. Patients and caregivers complained about physical complaints (adverse events) during DR-TB treatment. Difficulty in hearing was mentioned due to injectable while other adverse events mentioned due to DR-TB medications were difficulty in vision, pain in legs, skin colour darkening, nausea and vomiting during treatment.

*“The injections that I received, I have buzz in my ears. My hearing power has decreased.” (Patient 1; 15 year female)*.

“She used to get PAS, whenever she used to have that medicine she used to vomit it out. So I had to mix it with something and make her eat.” (Guardian of 15 year female).

*1*.*5*. *Drug compounding*. Caregivers of children and healthcare providers felt drug compounding was a tedious and complicated process for caregivers. Though training was provided to the caregivers, healthcare providers acknowledged the challenges faced by caregivers in process of preparing accurate dose for children each time.

*“It was difficult… I had to clean hands, wear gloves, then take the medicines… to prepare her medicines, one of them, I had to break into two, one part I used to give and other had to be thrown away.” (Guardian of 3 year old female)*.

“Drug compounding is a challenge because we do not have paediatric formulations. It’s too much for the mother to prepare each dose.” (Healthcare provider 6).

#### 2) Emotional trauma

*2*.*1*. *Long treatment*. The participants felt that the two-year treatment duration for DR-TB was too long. Six children and adolescents had previous TB treatment episodes and now reported treatment fatigue.

*“I spent 4 years with TB*. *I wanted to study more*, *become a teacher*. *This disease has taken important years of my life*.*”* (Patient 1, 15 year female).

*2*.*2*. *Adverse event*. *Skin darkening*. Skin colour discoloration due to Clofazamine was mentioned as the most stressful adverse event for patient and caregivers. It hampered patients’ motivation and contributed to self-stigma.

*“Her colour has darkened due to medicine, when we will start looking for match (groom for marriage), people will ask what has happened to her.” (Guardian of 15 year female)*.

“It is difficult for them (patients) to accept the way they look… colour of skin… they feel sad that they cannot talk to other friends, sometimes they go into depression.” (Healthcare provider 4).

*2*.*3*. *Anger and anxiety during treatment*. Anger and irritability was mentioned as an adverse event due to DR-TB medication (Cycloserine), while patients and caregivers were also anxious about cure of the patient. Many of the patients had previous TB episodes, and they were irritated and sad, why they had TB again; and now more severe form of disease. The anxiousness however, reduced with due course of treatment when they completed treatment.

*“I used to be very angry about the treatment. I used to hate the medicines.” (Patient 2, 18 year female)*.

“I was anxious that how will my child have these medicines for 2 long years, the duration was a lot for me. After the treatment started, I had no complaints.” (Guardian of 12 year male).

*2*.*4*. *Loneliness and disrupted social life*. Participants mentioned that DR-TB disease treatment led to missing school, college and playing with friends. Patients often had to stay indoors during treatment. Caregivers also could not leave the city during treatment and attend family events. Many caregivers lived with their child separate from their family, with the fear of TB transmission to others.

*“I used to love going out. I liked to go to school. I liked talking to friends. I used to get bored sitting at home.” (Patient 3; 16 year female)*.

“So I never used to go out anywhere. I used to think if I go out somewhere and I cannot come back home at night due to transport cancellation, bad weather, my child will miss the dose. With that fear, I never used to go anywhere far from house.” (Guardian of 3 year female).

*2*.*5*. *Multiple TB patients in family*. A majority of patients had their caregiver either suffering from TB or another family member who had or was currently suffering from TB. It was difficult for the caregiver to provide support to multiple members of family suffering from TB.

*“It is difficult for the mother to support the treatment of 3 children with different age group and different treatment. It’s too much of disease burden in the family to take care.” (Health care provider 8)*.

“Yes it was difficult, because I was also on treatment; it was difficult to maintain her health. (Guardian of 3 year old).

#### 3) Unavailable social support

*3*.*1*. *Girl child*. Healthcare providers mentioned that female children did not seem to receive equal care as male children, but caregivers and patients did not describe that. Caregivers were concerned about future of girl child after treatment, especially about her marriage. They did not prefer to disclose about DR-TB disease to prospective groom’s family.

*“For girl child many families do not pay attention to their treatment or timely food… the family doesn’t give 100% effort.” (Healthcare provider 1)*.

“I am also worried about my girl’s marriage, we can’t tell others that she had TB… it will be difficult to get her married.” (Guardian of 15 year female).

*3*.*2*. *Blame or shame*. Patients and caregivers expressed that they were either blamed for spreading the disease or they themselves had the guilt about this. The blame/shame of disease transmission reduced their interaction with other family members, and they were forced to stay alone during DR-TB treatment.

*“I stayed with my extended family, my uncle, aunt, cousins, grandmother. After my illness, my sister also got TB with me. She complained why I was brought from my village. She said she got TB from me, because of me her life finished.” (Patient 1; 15 year female)*.

“I was given treatment for MDR-TB 5–6 years back, when my youngest one (child) was born. He was also given treatment for 6 months so that he doesn’t have TB. When I got to know that my (two) children have illness, I thought I should go somewhere and die.” (Guardian of 8 year male).

*3*.*3*. *Discrimination in community*. Low awareness about TB in family and neighbourhood contributed to discrimination and stigma for patients in the community (including schools). Caregivers and patients mentioned that patients went to school without masks fearing discrimination.

*“My neighbours knew I had TB, so they used to tell everyone that they should stay away from me else I will spread the disease to them. I was asked not to sit outside home; they used to scold me that don’t come outside.” (Patient 1, 15 year female)*.

“… they asked me to wear mask in school, but you know, how can I wear it. They will ask what happened.” (Patient 3, 16 year female).

*3*.*4*. *Expensive treatment*. Though MSF Clinic provided DR-TB treatment free of cost, patients and caregivers mentioned about expenses incurred in previous TB treatment(s) which led to significant financial loss to the families.

“I took medicine for 2 months.. but the medicine was very expensive. My father told doctor that he cannot afford the expensive drugs.. so the doctor advised to go to municipality (government) hospital..” (Patient 1, 15 year female).

#### 4) Non-adapted healthcare services

*4*.*1*. *Minimum conversation with provider*. Patients complained about apathetic attitude of some healthcare providers. The patients and guardians expressed concern that few doctors did not communicate with them directly about their illness.

*“Doctors in hospital sometimes do not talk to us nicely… they keep us away from them. They say, don’t sit with other patients.” (Patient 5, 19 year male)*.

“Hospitals do not explain well… they do not talk well. Patients are asked to stay far from doctor… they are often angry on patients.” (Guardian of 8 year male).

*4*.*2*. *Irregular feedback during treatment*. Participants reported that the medical consultations during follow up were mainly restricted to adverse events management. No attention was given during follow up if they did not have adverse events.

*“Doctors should talk to patients first with care and emotion. They should not give stress to patients, asking why you are not taking medicine.” (Patient 5; 19 year male)*.

*“Feedback is only taken when they have adverse events*. *Yes*, *we miss taking feedback*. *We should ask them regularly after every 2 month*, *so that they feel they are spoken to*.*” (Healthcare provider 2)*.

*4*.*3*. *Inexistent age-adapted counselling tools*. Healthcare providers mentioned that child/adolescent friendly spaces and age-appropriate counselling tools were required for improved care for children and adolescents during DR-TB treatment. The patients and caregivers mentioned that they wanted more information about the DR-TB disease and treatment for them and their families.

*“We need to make audio-video or cartoons (about TB) for younger age group: 3–10 years” (Healthcare provider 5)*.

“They should explain what drugs is given, what will be the side effects. If we know it before, then we will not feel scared.” (Patient 1, 15 year female).

*4*.*4*. *Difficult adherence checklist*. According to participants, completion of paper-based monthly adherence monitoring tool was a tedious process and they (patients or caregivers) acknowledged that the patients often forgot to mark it at the time of medications. Healthcare providers mentioned they did not push patients or caregivers for completing the checklist, as they feared they would lose trust and will not confide their issues in healthcare providers.

*“I did not like filling it but my parents used to say, so I used to fill it up.” (Patient 2, 18 year female)*.

“Yes I received the treatment calendar to tick when I give medicines to her. But I could not completely follow it. I tried for few weeks, but the child used to tear it away or drop something on it.” (Guardian of 3 year female).

### Children and adolescents: Varied experiences

For younger children age-group (0–5 years), difficulties in compounding of drugs were noted while adolescents (10–19 years) shared experiences around disruption in social life due to disease and treatment. Few experiences of the older children (6–10 years) and their caregivers were similar to younger children (non-palatable pills, painful injectable, difficulties in drug compounding), while other experiences resembled to adolescents (unable to attend schools, meet friends). The adherence issues and treatment interruption were commonly reported in adolescents than children during DR-TB treatment. The healthcare providers highlighted the decision of treatment (regimen or provider) relied heavily on the family/caregiver of the children and adolescents.

*“In adolescents, we see this issue, patients going to native place/wedding/fair; they want to be there in all festivities. But in paediatric age group, patients do not miss doses; the caregivers have postponed their visit. Parents make sure that the treatment should be continuous.” (Healthcare provider 2)*.

“For these children, the power decision lies with the parent.. so even if the child is comfortable.. parent thinks otherwise.. we have to convince the parents a lot.” (Healthcare provider 1).

### Treatment fatigue and burnout in patients and caregivers

**Fig 2:**
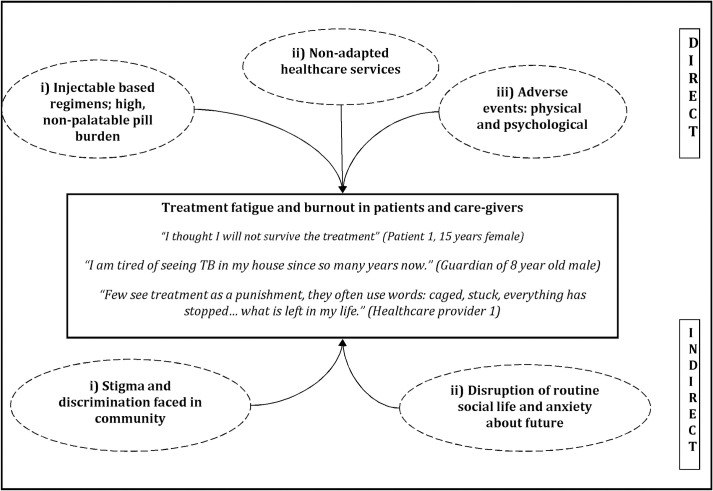
Factors of treatment fatigue and burnout in children and adolescents with DR-TB and their caregivers, Mumbai, India, 2019.

Treatment fatigue and burnout was noted in patients and caregivers due to multiple factors (Fig 2). The factors could be categorized as direct and indirect factors. The direct factors were related to DR-TB treatment and included i) Injectable based regimens; high, non-palatable pill burden, ii) Non-adapted healthcare services and iii) Adverse events: physical and psychological. The indirect factors were related to social experiences and quality of life experienced by patients and caregivers during DR-TB treatment of the patients. The indirect factors were i) Stigma and discrimination faced in community; ii) Disruption of routine social life and anxiety about future.

### Recommendations given by participants for developing child/ adolescent-friendly care

In addition to sharing the experiences and challenges during DR-TB in children and adolescents, the participants gave recommendations for developing child/adolescent-friendly care for the patients during DR-TB treatment. Changes in treatment regimens, healthcare services, care for patients and awareness about TB in family and community were suggested for improved care during DR-TB treatment. The recommendations for treatment regimen and healthcare services included non-injectable palatable medications, interaction with healthcare providers and information sharing, availability of peer-support platforms and patient friendly tools. Unbiased care and affection and TB awareness in families, schools and communities were also suggested. The list of recommendations including the participant quotes are presented in Table 1.

**Table 1:** Recommendations given by participants for developing child/adolescent-friendly care for patients during DR-TB treatment, Mumbai, India, 2019.

	Thematic domain	Recommendations by patients and caregivers	Recommendations from healthcare providers
***A***	***Treatment regimen and healthcare services***		
1	Non-injectable, palatable medications	*“Remove the injections from treatment*.*” (Guardian of 12 year male)*	*“Drug formulation is a big problem … we need dispersible tablets and syrups for children*.*” (Healthcare provider 6)*
2	Interaction with healthcare providers and information sharing	*“Doctor should tell them importance of medication*.*” (Patient 6; 18 year male)*	*“We need to make child friendly environment… so that they play*, *can talk to others*. *They always are with medicine at home and they get similar environment in the clinic*.*” (Healthcare provider 7) “For adolescents*, *we have to ask them how they want to take medicine; about timing of the medicine*. *They are happy if they are put in the driver seat*.*” (Healthcare provider 2)*
3	Availability of Peer-support platform	*“*.. *we all used to sit and talk about us*, *how we had our medicines*, *our side effects*. *So we used to feel that’s its just not us*, *other also have similar problem*.*” (Patient 1*, *15 year female)*	*“adolescents are part of adult support groups*, *that should not be the case*. *They have their own concerns and they are different than adults*.*” (Healthcare provider 1)*
4	Patient-friendly adherence tools	*“I have heard people use mobile phones for reminders during treatment but I do not know how to use mobile phone*.*” (Guardian of 3 year female)*	*“if there is a button on the medicine box which could mark that they have taken medicine/or parents have given medicine*, *that will help*, *because it happens that the medicine was kept in one box and paper was kept somewhere else*.*” (Healthcare provider 7)*
***B***	***Care for patients and awareness about TB in family and community***		
5	Family support with unbiased care and affection	*“We should try to give good lifestyle to child*, *even if they have lost weight and look skinny… we should not keep distances from them*, *we should talk to them*.*” (Guardian of 9 year female)*	*“We need an empowered buddy*. *When we have to start treatment for children*, *we have to start with the parents first*, *mother or father*, *whosoever comes forward as a buddy… so we have to prepare them*.*” (Healthcare provider 1)*
6	TB awareness in families, community (including schools)	*“People should get more messages that how TB is spread and not spread*. *Doctors*, *nurses should give messages to family*, *so that they are not scared*.*” (Patient 1*, *15 year female)*	*“If someone (family member) already knows about the disease*, *they understand the process and they are fast in follow up and maintain the follow up*.*” (Healthcare provider 3)*

## Discussion

Our study, one of the first in India to explore experiences of children and adolescents during DR-TB treatment; showed that children, adolescents and caregivers had a challenging treatment journey for DR-TB, which often disrupted their daily routine and future plans of life. The study revealed that the physical and emotional setbacks due to the long duration of DR-TB treatment, often coupled with previous TB experiences contributed to treatment fatigue and burnout in patients and their caregivers.

Previous studies reporting experiences of children and adolescents during DR-TB treatment included hospitalized patients, majority of whom were HIV co-infected [22,23]; while in our study, the participants were non-HIV and received DR-TB treatment on ambulatory basis. Though our study findings are in line with previous literature on paediatric DR-TB, our study contributes to the evidence around influence of families and communities in DR-TB treatment journey of children and adolescents. We believe our study from an urban high burden DR-TB slum setting provides important insights for improved treatment and care for DR-TB in children and adolescents.

Use of injectable agents was the most difficult part of treatment and all study participants (patients, guardians and healthcare providers) called for stopping the injectable drugs in DR-TB treatment. The injectable administration caused physical and emotional distress to the patients and guardians. This is in line with previous studies on DR-TB [3,12,24]. Injectable still continue to be part of the DR-TB regimen in many countries due to poor implementation of all-oral regimens in routine programmes and drug supply shortages. The WHO shorter regimen still includes injectable [25,26], which must be reconsidered.

The study results show the urgent need of palatable paediatric TB formulations for children. Guardians for paediatric patients complained that drug compounding was a difficult process and healthcare providers were uncertain about the quality for each prepared dose by the guardian, in line with previous studies [3,27,28]. TB programmes must provide access to already available paediatric formulations in routine programme and allocate budget for research and development activities and consider inclusion of children in future clinical trials [2], to develop palatable paediatric formulations for DR-TB.

The treatment fatigue and burnout for patients and caregivers reported in our study add to the evidence that treatment fatigue due to long, painful treatment is often reported among adolescents and adult patients [14,29]. The burnout in caregivers is escalated by their first-hand experience during their own DR-TB treatment journey and while supporting multiple DR-TB patients in one family.

The stigma related to DR-TB still exists in the community and affect the life during DR-TB treatment of patients [22]. In particular, the darkening of skin as an adverse event due to Clofazamine are emotionally tiring for patients and caregivers, that hamper their social interactions with friends, community and contribute to internalized stigma [13], more so in case of adolescents. Caregivers are anxious about marriage of their female child. Age-specific counselling tools must be developed and repeat sessions must be organized during treatment follow up. Group counselling sessions and support group meetings could be organized to discuss marriage concerns. To tackle stigma in community against DR-TB in general, mass awareness campaigns could be organized which will also aid in limiting TB transmission [23].

Child or adolescent friendly spaces are urgently needed for improved care. Participants mentioned that empathy was often missing in consultations. Patients felt they were only recipients of one-way communication from healthcare providers. Continuous dialogue and shared ownership during treatment follow up, especially in adolescents may help in improving care [26].

Family or peer support was identified as the cornerstone for treatment success in patients [23,29]. An ‘empowered buddy’ provided support to the patient during their entire journey of treatment. However, the DR-TB programme must acknowledge the family rituals (cultural practices, participation in festivals and interaction with faith healers) while designing the treatment routine and follow up visits [23,30].

Patients and caregivers complained about cumbersome and inconvenient process of completing DR-TB treatment monitoring tools. Though new ideas of novel techniques of monitoring tool are warranted, it must address the context specific hurdles related to usage [24]. Involvement of patients and caregivers in developing monitoring tools will help in development of an effective monitoring tool for patients.

Our study had few limitations. The sample was purposive and may not represent all adolescents, guardians and healthcare-providers involved in DR-TB treatment. None of the patients were HIV co-infected, thus the study results may not correspond to HIV DR-TB co-infected children and adolescents. Though the study had purposive sampling, the researchers included older adolescents for interviews due to operational feasibility, thus the experiences of older adolescent influence the results of the study. The interviewer was employee of the organisation, however was neither involved in direct care of patients nor was part of the project team. However, we do admit there could be social desirability bias in their responses. Although the study had multiple limitations, we believe our study has provided first-hand experiences of patients, caregivers and healthcare providers about DR-TB treatment journey of children and adolescents in high DR-TB burden setting.

## Conclusion

In conclusion, treatment and care for DR-TB in children and adolescent age groups need tailored socio-psychological approach along with medical care. The TB programmes must consider the patient and family as one unit when designing the package of care for paediatric DR-TB. The direct or indirect emotional, social and financial support when offered with treatment will help to minimize disruption of social life and burnout in patients and caregivers during DR-TB treatment.

## Supporting information

S1 Appendix Interview guides (in English and local language-Hindi) used for in-depth interviews of participants, Mumbai, India, 2019. (DOC)
