# Correction: Challenging drug-resistant TB treatment journey for children, adolescents and their care-givers: A qualitative study

**DOI:** 10.1371/journal.pone.0299482

**Published:** 2024-02-21

**Authors:** Mrinalini Das, Taanya Mathur, Shilpa Ravi, Augusto C. Meneguim, Aparna Iyer, Homa Mansoor, Stobdan Kalon, Farah Naz Hossain, Shrikala Acharya, Gabriella Ferlazzo, Petros Isaakidis, Harshad P. Thakur

There are errors in the Funding and Competing Interests statements. The correct statements are:

**Funding:** Médecins Sans Frontières (MSF) provided support in the form of salaries for MD, TM, SR, ACM, AI, HM, SK, FNH, GF, and PI. The specific roles of these authors are articulated in the ‘author contributions’ section. MSF programmatic funding covered all other costs of the study. The funder was involved in study design, data collection and analysis, decision to publish, and preparation of the manuscript.

**Competing Interests:** The authors have read the journal’s policy and have the following competing interests to declare: Médecins Sans Frontières (MSF) provided support in the form of salaries for MD, TM, SR, ACM, AI, HM, SK, FNH, GF, and PI. This does not alter our adherence to *PLOS ONE* policies on sharing data and materials. There are no patents, products in development, or marketed products associated with this research to declare.
