# Supplementary material for: Challenging drug-resistant TB treatment journey for children, adolescents and their care-givers: A qualitative study
Source: PLoS One. 2021 Mar 10;16(3):e0248408. doi: 10.1371/journal.pone.0248408 (PMC7946226; doi:10.1371/journal.pone.0248408)
Supplement: S1 Appendix — (DOC) [file pone.0248408.s001.doc]

**Challenging drug-resistant TB treatment journey for children, adolescents and their care-givers: A qualitative study**

**S1 Appendix. Interview guides (in English and local language-Hindi) used for in-depth interviews of participants, Mumbai, India, 2019**

**Annexure-1a**

## Interview guide

## (Parents/Guardians of Child and adolescents with Drug-resistant TB)

Date of interview:

Participant ID:

Interview start / end time:

Name of the interviewer:

After a brief introduction to the participant (parent/guardian of child and adolescents with Drug-resistant TB) regarding the purpose of the interview, the PI will take informed written consent for the interview. Written informed consent will also be requested for audio recording.

1. Your child is being treated for Drug-resistant Tuberculosis. Please tell me about how you found out your child had DR-TB? (Probe- diagnostic test, time taken to confirm, hospital visits duration)
2. Could you please tell me about your child’s treatment? (Probe- Drugs, effect on school attendance, visit to hospital, adverse events)
3. Could you please share your experience related to how your child feels about his/her illness and treatment? (Probe- determined, stigma, anxiety, denial)
4. Please share your experience how you feel the DR-TB treatment has affected your child’s daily life routine activities? If yes, how has it affected his/her routine?
5. How do you support your child in taking his/her medication on time? (Probe- during school, vacation, festivals, marriages)
6. Can you describe events when your child missed taking medications? What did you do then? (Probe- during examination, during fasting, parent’s illness, guests at home)
7. Can you share if your child refused to take further medication after he/she had vomiting/dizziness due to medications? How did you convince him/her for continuing treatment?
8. How do you feel about the support provided by healthcare workers for helping your child to take medications regularly, on time? Can you share your views on how healthcare workers explain you/your child about importance of taking medicine regularly? Would you like to share some instances
9. Can you please share what you feel, how the clinical team can support you/ your child/other children of same age-group so that your child/other children of same age-group with DR-TB take medicines on time and complete treatment.
10. Can you please tell me if you have any ideas/suggestions that can be done to make it easier for children and adolescents to take medications, adhere and complete his/her treatment?
11. What advice would you give to another person whose child was just diagnosed with DR-TB?
12. Please feel free to add any comment that you would like to make

**Annexure-1b (Hindi translation)**

**साक्षात्कार गाइड**

**(डीआर-टीबी बच्चे और डीआर-टीबी वाले किशोर के माता-पिता / अभिभावक)**

साक्षात्कार की तिथि:

प्रतिभागी आईडी:

साक्षात्कार प्रारंभ / समाप्ति समय:

साक्षात्कारकर्ता का नाम:

साक्षात्कार के उद्देश्य के बारे में प्रतिभागी (बच्चे के माता-पिता / अभिभावक और ड्रग-प्रतिरोधी टीबी के साथ किशोरों) के संक्षिप्त परिचय के बाद, पीआई साक्षात्कार के लिए लिखित सहमति देगा। ऑडियो रिकॉर्डिंग के लिए लिखित सूचित सहमति भी मांगी जाएगी।

1. आपके बच्चे का इलाज डीआर-टीबी के लिए किया जा रहा है। कृपया मुझे इस बारे में बताएं कि आपको कैसे पता चला कि आपके बच्चे को डीआर-टीबी है? (जांच- ​​परीक्षण, पुष्टि के लिए लिया गया समय, अस्पताल का दौरा अवधि)
2. क्या आप मुझे अपने बच्चे के इलाज के बारे में बता सकते हैं? (जांच- दवाओं, स्कूल में उपस्थिति पर प्रभाव, अस्पताल का दौरा, प्रतिकूल घटनाएँ)
3. क्या आप कृपया अपने अनुभव को साझा कर सकते हैं कि आपका बच्चा अपनी बीमारी और उपचार के बारे में कैसा महसूस करता है? (जांच- निर्धारित, कलंक, चिंता, इनकार)
4. कृपया अपना अनुभव साझा करें कि आपको कैसा लगता है DR-TB उपचार से आपके बच्चे की दैनिक जीवन की गतिविधियाँ प्रभावित हुई हैं? यदि हाँ, तो इससे उसकी दिनचर्या कैसे प्रभावित हुई है?
5. आप अपने बच्चे को समय पर उसकी दवा लेने में कैसे मदद करते हैं? (जांच- स्कूल, छुट्टी, त्योहार, विवाह के दौरान)
6. क्या आप उन घटनाओं का वर्णन कर सकते हैं जब आपका बच्चा दवाएँ लेने से चूक गया हो? फिर आपने क्या किया? (जांच- परीक्षा के दौरान, उपवास के दौरान, माता-पिता की बीमारी, घर पर मेहमान)
7. क्या आप साझा कर सकते हैं यदि आपके बच्चे ने दवाइयों के कारण उल्टी / चक्कर आना बंद कर दिया है? उपचार जारी रखने के लिए आपने उसे कैसे मना लिया?
8. आप अपने बच्चों को नियमित रूप से समय पर दवाएँ लेने में मदद करने के लिए स्वास्थ्य सेवा कार्यकर्ताओं द्वारा दिए गए समर्थन के बारे में कैसा महसूस करते हैं? क्या आप इस बारे में अपने विचार साझा कर सकते हैं कि स्वास्थ्यकर्मी आपको / आपके बच्चे को नियमित रूप से दवा लेने के महत्व के बारे में कैसे समझाते हैं? क्या आप कुछ उदाहरण साझा करना चाहेंगे
9. आप क्या महसूस करते हैं, टीम आपको / आपके बच्चे / एक ही आयु-वर्ग के अन्य बच्चों का समर्थन कैसे कर सकती है ताकि आपका बच्चा / अन्य आयु-वर्ग के बच्चे DR-TB के साथ समय पर दवाएँ लें
10. क्या आप मुझे बता सकते हैं कि क्या आपके पास कोई विचार / सुझाव है जो बच्चों और किशोरों के लिए दवाएँ लेने, उनका पालन करने और उनके उपचार को पूरा करने के लिए आसान बनाने के लिए किया जा सकता है?
11. आप किसी अन्य व्यक्ति को क्या सलाह देंगे जिसके बच्चे को DR-TB का पता चला था?
12. कृपया कोई भी टिप्पणी जोड़ने के लिए स्वतंत्र महसूस करें

## Annexure 2a

## Interview guide

## [Adolescents (aged 10-19 years) with DR-TB]

Date of interview:

Participant ID:

Interview start / end time:

Name of the interviewer:

After a brief introduction to the participant regarding the purpose of the interview, the PI will take informed written consent for the interview. Written informed consent will also be requested for audio recording. For adolescents aged 10-17 years, assent of adolescent and consent of guardian/parent will be taken.

1. You are being treated for Drug-resistant Tuberculosis. Can you share your thoughts about how you got to know that you are suffering from DR-TB? (Probe- diagnostic test, time taken to confirm, hospital visits duration)
2. Can you share how you feel about your disease (Probe- determined, stigma, anxiety, and denial) and treatment? (Probe- Drugs, effect on school/college/job attendance, visit to hospital, adverse events)
3. Can you please share incidence if you felt the DR-TB treatment has affected your daily life routine activities? If yes, how has it affected your routine?
4. We understand taking complete medications will help in treating the DR-TB disease. How do you continue taking medication on time? (Probe- during school/college/work, vacation, festivals, marriages)
5. Has there been incidence if you missed taking medications? What did you do then? (Probe- during examination, during fasting, family member’s illness, urgent travel, guests at home)
6. Can you share if you ever thought to stop taking further medication after you had vomiting/dizziness due to medications? Who helped you in continuing treatment after that episode?
7. How do you feel about the support provided by healthcare workers for helping you take medications regularly, on time? Can you share your views on how healthcare workers explain you about importance of taking medicine regularly? Would you like to share some instances
8. Can you please share what you feel, how the clinical team can support you/other patients with DR-TB of your age so that they take medicines on time and complete treatment.
9. Can you please tell me if you have any ideas/suggestions that can be done to make it easier for children and adolescents of your age to take medications, adhere and complete his/her treatment.
10. Please feel free to add any comment that you would like to make

**Annexure-2b (Hindi translation)**

**साक्षात्कार गाइड**

**(DR-TB के साथ किशोर (10-19 वर्ष की आयु)**

साक्षात्कार की तिथि:

प्रतिभागी आईडी:

साक्षात्कार प्रारंभ / समाप्ति समय:

साक्षात्कारकर्ता का नाम:

साक्षात्कार के उद्देश्य के बारे में प्रतिभागी से संक्षिप्त परिचय के बाद, पीआई साक्षात्कार के लिए लिखित सहमति देगा। ऑडियो रिकॉर्डिंग के लिए लिखित सूचित सहमति भी मांगी जाएगी। 10-17 वर्ष की आयु के किशोरों के लिए, अभिभावक / माता-पिता की सहमति ली जाएगी।

1. आपको दवा प्रतिरोधी तपेदिक के लिए इलाज किया जा रहा है। क्या आप अपने विचारों को साझा कर सकते हैं कि आपको कैसे पता चला कि आप DR-TB से पीड़ित हैं? (जांच- नैदानिक ​​परीक्षण, पुष्टि के लिए लिया गया समय, अस्पताल का दौरा अवधि)
2. क्या आप साझा कर सकते हैं कि आप अपनी बीमारी के बारे में कैसा महसूस करते हैं (जांच-निर्धारित, कलंक, चिंता और इनकार) और उपचार? (जांच- ड्रग्स, स्कूल / कॉलेज / नौकरी की उपस्थिति पर प्रभाव, अस्पताल का दौरा, प्रतिकूल घटनाएँ)
3. क्या आप घटना को साझा कर सकते हैं यदि आपको लगा कि डीआर-टीबी के उपचार ने आपके दैनिक जीवन की गतिविधियों को प्रभावित किया है? यदि हाँ, तो यह आपकी दिनचर्या को कैसे प्रभावित करता है?
4. हम समझते हैं कि पूर्ण दवाएं लेने से डीआर-टीबी रोग का इलाज करने में मदद मिलेगी। आप समय पर दवा लेना कैसे जारी रखते हैं? (जांच- स्कूल / कॉलेज / काम, छुट्टी, त्योहार, विवाह के दौरान)
5. क्या दवाएं लेने से चूक गए हैं? फिर आपने क्या किया? (जांच- परीक्षा के दौरान, उपवास के दौरान, परिवार के सदस्य की बीमारी, तत्काल यात्रा, घर पर मेहमान)
6. क्या आप साझा कर सकते हैं अगर आपने कभी दवाइयों के कारण उल्टी / चक्कर आने के बाद आगे की दवा लेना बंद करने के बारे में सोचा? उस एपिसोड के बाद निरंतर उपचार में आपकी मदद किसने की?
7. समय पर आपको नियमित रूप से दवाएँ लेने में मदद करने के लिए स्वास्थ्य कर्मियों द्वारा दिए गए समर्थन के बारे में आप कैसा महसूस करते हैं? क्या आप इस बारे में अपने विचार साझा कर सकते हैं कि स्वास्थ्यकर्मी आपको नियमित रूप से दवा लेने के महत्व के बारे में कैसे समझाते हैं? क्या आप कुछ उदाहरण साझा करना चाहेंगे
8. क्या आप कृपया महसूस कर सकते हैं कि आप क्या महसूस करते हैं, कैसे ​​टीम आपकी उम्र के DR-TB के साथ / अन्य रोगियों का समर्थन कर सकती है ताकि वे समय पर दवाएँ लें और पूरा इलाज करें।
9. क्या आप मुझे बता सकते हैं कि क्या आपके पास कोई विचार / सुझाव है, जो आपके बच्चों और किशोरों के लिए दवाएँ लेने, उनका पालन करने और उनके उपचार को पूरा करने के लिए आसान हो सकता है।
10. कृपया कोई भी टिप्पणी जोड़ने के लिए स्वतंत्र महसूस करें

## Annexure 3a

## Interview guide (Health Care Provider)

Date of interview:

Participant ID:

Interview start / end time:

Name of the interviewer:

After a brief introduction to the participant regarding the purpose of the interview, the PI will take informed written consent for the interview. Written informed consent will also be requested for audio recording

1. You are providing clinical care to children and adolescents with DR-TB. How do you feel about the DR-TB diagnostic techniques for children and adolescents (multiple tests, time taken, clinical diagnosis, EPTB)
2. According to you, how do they feel about the illness (Probe- determined, stigma, anxiety, denial) and treatment (Probe- Drugs, effect on school/college/job attendance, visit to hospital, adverse events)?
3. Can you please share your views regarding involvement of their family members, community, friends, and school in continuation of treatment?
4. How do you assess treatment adherence in children and adolescents? What do you use? Could you please share the details
5. What are the instances when children miss medicine doses or refuse continuation of treatment? Can you explain some examples (Probe- during examination, during fasting, family member’s illness, urgent travel, guests at home)? How are these different for adolescents?
6. Have children/ adolescents reported to you about missing doses? What did you do then?
7. Can you share if the patients/guardians interrupted treatment after patients had vomiting/dizziness due to medications? How did you convince them in continuing treatment after that episode?
8. How do you educate them about adherence of treatment and support them to take medicines regularly, on time? What additional support you provide to children and adolescents for treatment adherence in comparison to adult patients? Would you like to share some instances
9. Are you comfortable with existing adherence monitoring tools for children? Is it different than monitoring for adults? Please share your views.
10. What are the challenges you encounter in maintaining the patients on treatment? Can you share some examples how you convinced treatment interrupters to return for treatment.
11. Can you please share what you feel, how the clinical team can support children and adolescents with DR-TB towards a patient centred approach, so that they take medicines on time and complete treatment.
12. Can you please tell me if you have any ideas/suggestions that can be done to make it easier for children and adolescents with DR-TB to take medications, adhere and complete his/her treatment.
13. Please feel free to add any comment that you would like to make

**Annexure-3b (Hindi translation)**

**साक्षात्कार गाइड (स्वास्थ्य सेवाएं देने वाला)**

साक्षात्कार की तिथि:

प्रतिभागी आईडी:

साक्षात्कार प्रारंभ / समाप्ति समय:

साक्षात्कारकर्ता का नाम:

साक्षात्कार के उद्देश्य के बारे में प्रतिभागी से संक्षिप्त परिचय के बाद, पीआई साक्षात्कार के लिए लिखित सहमति देगा। ऑडियो रिकॉर्डिंग के लिए लिखित सूचित सहमति भी मांगी जाएगी

1. आप बच्चों और किशोरों को डीआर-टीबी के साथ नैदानिक ​​देखभाल प्रदान कर रहे हैं। आप बच्चों और किशोरों के लिए DR-TB नैदानिक ​​तकनीकों के बारे में कैसा महसूस करते हैं (एकाधिक परीक्षण, समय लिया, नैदानिक ​​निदान, EPTB)
2. आपके अनुसार, वे बीमारी के बारे में कैसा महसूस करते हैं (जांच - निर्धारित, कलंक, चिंता, इनकार) और उपचार (जांच- ड्रग्स, स्कूल / कॉलेज / नौकरी की उपस्थिति पर प्रभाव, अस्पताल का दौरा, प्रतिकूल घटनाएं)?
3. क्या आप उपचार की निरंतरता में अपने परिवार के सदस्यों, समुदाय, दोस्तों और स्कूल की भागीदारी के बारे में अपने विचार साझा कर सकते हैं?
4. आप बच्चों और किशोरों में उपचार के पालन का आकलन कैसे करते हैं? तुम क्या इस्तेमाल करते हो? क्या आप कृपया विवरण साझा कर सकते हैं
5. ऐसे कौन से उदाहरण हैं जब बच्चों को दवा याद आती है या उपचार जारी रखने से मना करते हैं? क्या आप कुछ उदाहरण (जांच- परीक्षा के दौरान, उपवास के दौरान, परिवार के सदस्य की बीमारी, तत्काल यात्रा, घर पर मेहमान) के बारे में बता सकते हैं? किशोरों के लिए ये कैसे भिन्न हैं?
6. क्या बच्चों / किशोरों ने आपको गुम खुराक के बारे में सूचना दी है? फिर आपने क्या किया?
7. क्या आप साझा कर सकते हैं कि दवाइयों के कारण रोगियों को उल्टी / चक्कर आने के बाद रोगियों / अभिभावकों ने इलाज बाधित किया? आपने उस एपिसोड के बाद निरंतर उपचार में उन्हें कैसे मना लिया?
8. आप उपचार के पालन के बारे में उन्हें कैसे शिक्षित करते हैं और समय पर नियमित रूप से दवा लेने के लिए उनका समर्थन करते हैं? वयस्क रोगियों की तुलना में उपचार के पालन के लिए आप बच्चों और किशोरों को क्या अतिरिक्त सहायता प्रदान करते हैं? क्या आप कुछ उदाहरण साझा करना चाहेंगे
9. क्या आप बच्चों के लिए मौजूदा पालन निगरानी उपकरणों के साथ सहज हैं? क्या यह वयस्कों के लिए निगरानी से अलग है? कृपया अपने विचार साझा करें।
10. उपचार के दौरान मरीजों को बनाए रखने में आपके सामने क्या चुनौतियाँ हैं? क्या आप कुछ उदाहरण साझा कर सकते हैं कि आपने उपचार के लिए उपचार अवरोधकों को कैसे मना लिया।
11. क्या आप मुझे बता सकते हैं कि आप क्या महसूस करते हैं, कैसे नैदानिक ​​टीम एक रोगी केंद्रित दृष्टिकोण के लिए DR-TB के साथ बच्चों और किशोरों का समर्थन कर सकती है, ताकि वे समय पर दवाएँ लें और पूरा इलाज करें।
12. क्या आप मुझे बता सकते हैं कि क्या आपके पास कोई विचार / सुझाव है जो दवाइयों का सेवन करने, पालन करने और उसके उपचार को पूरा करने के लिए बच्चों और किशोरों के लिए डीआर-टीबी को आसान बनाने के लिए किया जा सकता है।
13. कृपया कोई भी टिप्पणी जोड़ने के लिए स्वतंत्र महसूस करें

## Annexure 4

## Interview guide (Programme managers)

Date of interview:

Participant ID:

Interview start / end time:

Name of the interviewer:

After a brief introduction to the participant regarding the purpose of the interview, the PI will take informed written consent for the interview. Written informed consent will also be requested for audio recording

1. How do you feel about the DR-TB diagnostic techniques for children and adolescents (multiple tests, time taken, clinical diagnosis, EPTB)
2. According to you, how do the patients and their family members feel about the illness (Probe- determined, stigma, anxiety, and denial) and treatment (Probe- Drugs, effect on school/college/job attendance, visit to hospital, adverse events)?
3. Can you please share your views regarding involvement of children and adolescent’s family members, community, friends, and school in continuation of treatment?
4. What are the instances when children miss medicine doses or refuse continuation of treatment? Can you explain some examples (Probe- during examination, during fasting, family member’s illness, urgent travel, guests at home)? How are these different for adolescents?
5. Please tell me about any reports that you may have received regarding children/ adolescents missing doses or interrupting treatment? If so, what measures were taken for that?
6. How do you feel about treatment literacy and education about adherence of treatment is provided to patients and family members? What additional support the programme provides to children and adolescents for treatment adherence in comparison to adult patients? Would you like to share some instances
7. What are the challenges you feel are encountered by the programme in maintaining the patients on treatment? Can you share some examples when treatment providers convinced treatment interrupters to return for treatment
8. Can you please share what you feel, how the clinical team can support children and adolescents with DR-TB towards a patient centred approach, so that they take medicines on time and complete treatment.
9. Can you please tell me if you have any ideas/suggestions that can be done to make it easier for children and adolescents with DR-TB to take medications, adhere and complete his/her treatment.
10. Please feel free to add any comment that you would like to make

END
